# Chimeras in Merlot grapevine revealed by phased assembly

**DOI:** 10.1186/s12864-023-09453-8

**Published:** 2023-07-14

**Authors:** V. Sichel, G. Sarah, N. Girollet, V. Laucou, C. Roux, M. Roques, P. Mournet, L. Le Cunff, P.F. Bert, P. This, T. Lacombe

**Affiliations:** 1 UMR AGAP Institut, Univ Montpellier, CIRAD, INRAE, Institut Agro, Montpellier, F-34398 France; 2 EGFV, Université de Bordeaux, Bordeaux-Sciences Agro, INRAe, ISVV, 210 Chemin de Leysotte, F-33882 Villenave d’Ornon, France; 3 Institut Français de la Vigne et du Vin, Montpellier, F-34398 France; 4 UMT Geno-Vigne®, IFV-INRAE-Institut Agro, Montpellier, F-34398 France; 5 UMR AGAP Institut, CIRAD, Montpellier, F-34398 France

**Keywords:** Chimera, Hifi sequencing, Phased assembly, Whole genome, *Vitis vinifera*

## Abstract

Chimerism is the phenomenon when several genotypes coexist in a single individual. Used to understand plant ontogenesis they also have been valorised through new cultivar breeding. Viticulture has been taking economic advantage out of chimeras when the variant induced an important modification of wine type such as berry skin colour. Crucial agronomic characters may also be impacted by chimeras that aren’t identified yet. Periclinal chimera where the variant has entirely colonised a cell layer is the most stable and can be propagated through cuttings. In grapevine, leaves are derived from both meristem layers, L1 and L2. However, lateral roots are formed from the L2 cell layer only. Thus, comparing DNA sequences of roots and leaves allows chimera detection. In this study we used new generation Hifi long reads sequencing, recent bioinformatics tools and trio-binning with parental sequences to detect periclinal chimeras on ‘Merlot’ grapevine cultivar. Sequencing of cv. ‘Magdeleine Noire des Charentes’ and ‘Cabernet Franc’, the parents of cv. ‘Merlot’, allowed haplotype resolved assembly. Pseudomolecules were built with a total of 33 to 47 contigs and in few occasions a unique contig for one chromosome. This high resolution allowed haplotype comparison. Annotation was transferred from PN40024 VCost.v3 to all pseudomolecules. After strong selection of variants, 51 and 53 ‘Merlot’ specific periclinal chimeras were found on the Merlot-haplotype-CF and Merlot-haplotype-MG respectively, 9 and 7 been located in a coding region. A subset of positions was analysed using Molecular Inversion Probes (MIPseq) and 69% were unambiguously validated, 25% are doubtful because of technological noise or weak depth and 6% invalidated. These results open new perspectives on chimera detection as an important resource to improve cultivars through clonal selection or breeding.

**Supplementary Information:**

The online version contains supplementary material available at 10.1186/s12864-023-09453-8.

## Background

### Chimera phenomenon

Individuals including cells with different genotypes are called chimeras or genetic mosaics. They are formed when a somatic genetic variation appears in a single cell in the meristem and is propagated through cell divisions. Occasionally the variation modifies a character of the plant and makes the chimera visible. This phenomenon called sporting was observed many centuries ago and has been fascinating scientists since then [1–3]. Chimeras can also induce variegation which is a very noticeable sporting type because it appears as a mosaic of colours in either leaves, flowers or fruits [4]. In 1907, Winkler was the first to use the term chimera while observing grafted plants [5]. Then, the investigation of variegation led Erwin Baur’s on the path of non-Mendelian inheritance [6]. Later, colchicine treatment on Datura seeds revealed periclinal chimeras [7] which allowed the understanding of cell lineages and ontogenesis of plant organs [8–10]. Indeed, two main types exist: sectorial and periclinal chimeras. The differences between those are the complete (periclinal) or incomplete (sectorial) colonisation of a cell layer by the somatic variation [11–13]. Periclinal chimeras are the most stable and can propagate by vegetative multiplication from cuttings [14]. Chimeras have already highly contributed to plant ontogenesis comprehension [10, 15]. In most cases these mutations are silent although some may modify the plant’s phenotype on important agronomic traits [16]. They can be used to study biosynthetic pathways [17] but should also be considered as an important source to improve current cultivars or breed new ones [18, 19].

Viticulture has already been taking economic advantage of grapevine chimeras by propagating the new phenotype as a new cultivar. For instance, genetic mosaics explain the origin and the evolution between cv. ‘Pinot Blanc’ and ‘Pinot Gris’ with a modification of berry skin colour [20, 21] and between several *teinturier* cultivars [22]. A somatic mutation in cv. ‘Meunier’, derived from ‘Pinot Noir’, was used to produce a microvine which strongly accelerates physiology, biology and genetics studies [23].

**Fig. 1:**
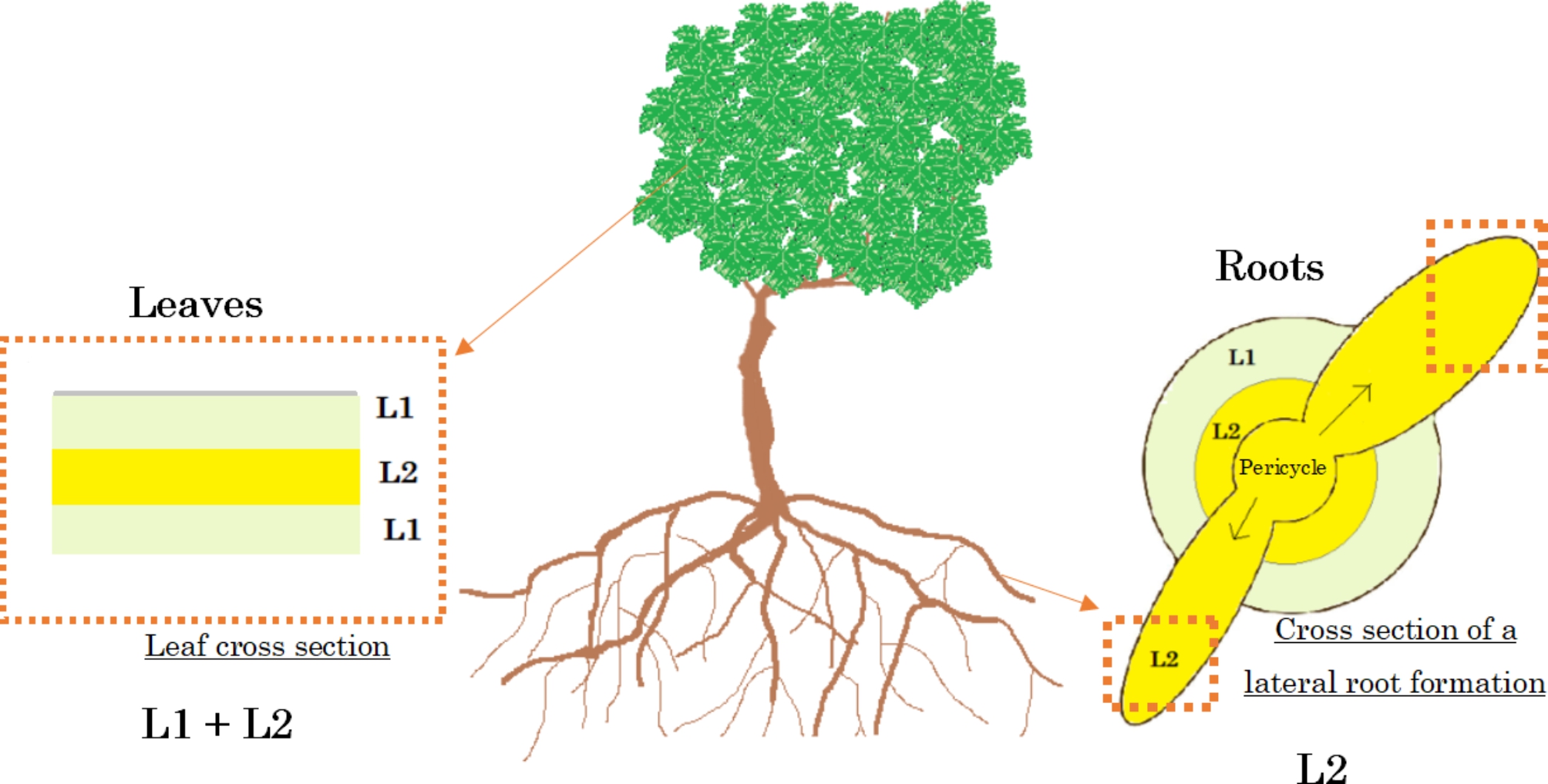
Cellular layers in grapevine roots and leaves. Schematic representation of a grapevine plant. Leaf and lateral root cross sections are enlarged in order to present the different cell layers present in both organs. Leaves are derived from both L1 and L2 meristem cell layers, while lateral roots are only formed out of the L2 layer

For these reasons, grapevine is a good example to study chimeras in woody plant species. While plant organs generally originate from three meristematic cell layers, grapevine organs come from only two functional cell layers (L1 and L2) in the apical meristem [12]. Leaves derived from L1 and L2 cell layers while other organs like gametophytic tissues and lateral roots originate from L2 cell layer only [12, 24]. Indeed, lateral roots are formed from the differentiation of the meristematic L2 cell layer (Fig. 1). Comparing the whole DNA sequence obtained through leaf samples (L1 + L2) with lateral roots samples (L2 only) could allow us to identify chimeras that may not be visible but play an important role in intra-varietal genetic diversity. In order to do this, a high quality whole genome sequence to accurately validate a chimera against sequencing mistakes is necessary. The assembly genome also needs to be resolved per haplotype to distinguish chimeras from grapevine heterozygosity.

### Whole genome new generation sequencing

The first grapevine whole genome sequencing was published in 2007 by the French-Italian consortium [25]. Since then, the ‘PN40024’ sequence obtained with a nearly homozygous inbred ‘Pinot Noir’ plant has been the reference genome for *Vitis sp.* The first version had 8X coverage and has been gradually updated through the 12x.v0, the 12x.v2 [26] and the most recent one is PN40024.v4 which has 40X coverage (European Nucleotide Archive - project PRJEB45423). Having a good quality whole genome reference has highly increased the understanding of *Vitis vinifera* genome but the nearly homozygous plant (PN40024 is homozygous on 93% of the genome) can hardly be considered as representative of cultivars used in grape production.

Short reads technology produces accurate genome sequencing, but because of the highly repetitive sequences of the grapevine genome they are difficult to assemble, therefore producing a whole reference genome with short reads can become very challenging [27]. Long reads technology has allowed massive improvement in genome assembly. The Falcon-Unzip phasing algorithm was very successful on Arabidopsis but had more difficulty with the grape cv. ‘Cabernet-Sauvignon’ sample because of the high rate of heterozygous position and the amount of repetitive sequences [28]. Purging treatment on haplotigs allowed to increase the assembly quality on the ‘Chardonnay’ sequence [29]. The ‘Carmenère’ phased assembly was also improved by optimising coverage, error correction, repeat masking methods and assembly parameters of FALCON-UNZIP [30]. Since then resolving haplotype phased assembly has become more accessible and numerous cultivars of the *Vitis* genus have been sequenced (Table 1).

**Table 1:** Whole grapevine genome sequences published until today:

Authors	Year	Technology	Grapevine genotype	Size (mbp)	Haplotigs size (mbp)	Coverage	N50 (kbp)	BUSCO
[25] Jaillon et al.	2007	Sanger	PN 40,024 (12x.v1)	487		8X	65.9	
[26] Canaguier et al.	2017	Sanger	PN 40,024 (12x.v2)			12X		
[28] Chin et al.	2017	PacBio RS II	Cabernet-Sauvignon	591		140X	72	80%
[29] Roach et al.	2018	PacBio RS II	Chardonnay	490		115X	935.8	95%
[30] Minio et al.	2019	PacBio RS II	Carmenère	622		115X	1 040	95%
[31] Girollet et al.	2019	PacBio RS II	*Vitis riparia*	500		225X	1 000	95%
[32] Massonnet et al.	2020	PacBio RS II PacBio Sequel II	*Muscadinia rotundifolia*	460	364	115X	4 761	97%
			*Vitis arizonica* b40-14	604	337	160X	1 536	96%
			*Vitis vinifera* subsp. *sylvestris* O34-16	678	252	85X	998	97%
			*Vitis vinifera* subsp. *sylvestris* DVIT3603.16	667	314	82X	2 661	97%
			*Vitis vinifera* subsp. *sylvestris* DVIT3603.07	663	234	61X	1 169	97%
			*Vitis vinifera* subsp. *sylvestris* DVIT3351.27	670	311	77X	1 778	97%
			Zinfandel	591	306	94X	1 062	97%
			Merlot	606	244	64X	810	94%
			Black Corinth 2.1	672	288	63X	1 113	97%
			Black Corinth seeded	650	357	75X	2 309	97%
			Cabernet-Sauvignon	449	444	147X	24 161	97%
[33] Zou et al.	2021	PacBio Sequel I	Carmenère	623	420	112x	1 039	97%
		PacBio Sequel I	Riesling	742	323	118x	2 970	98%

Each references, are sorted by authors and publication year. The sequencing technology used is specified as well as the genotype’s name. The following data allows to compare sequencing quality by: the total size of the genome and the haplotig size, the average coverage, the N50 value which means that half of the genome is formed with contigs bigger than this size and the percentage of gene detected from BUSCO analysis

Third generation long reads sequencing with high accuracy brings us into a new perspective of whole genome sequencing. Bioinformatics engineering is adapting to these new sequencing technologies, long reads haplotype assembly is now possible for diploids through tools like Hi-Canu and Hifiasm [34]. To increase accuracy and have a better chance to resolve haplotype phasing, trio-binning with parental sequences can be used to sort the child’s reads in two groups [35]. Up to now different techniques have led to chimera detection: (i) random amplified polymorphic DNA [36]; (ii) comparing phenotypes of regenerated plants from different cell layers [14]; (iii) comparing microsatellites markers in wood or roots tissues (L2) against leaves (L1 + L2) coming from the same plant [37]; (iv) flow cytometry measurements on pericarp and flesh fruit tissues in order to compare ploidy level between L1 and L2 [38]; (v) Real Time PCR on regenerated transgenic plants in order to evaluate the amount of chimeras and the uniformity of the transformation [39]; (vi) microsatellite (SSR) amplification by PCR when three alleles are found on one loci and confirmed by comparing different regenerated plants [40]; (vii) comparing DNA sequences obtained from different tissues dissection of leaf or berry skin (L1 + L2) against flesh or roots (L2 only) [20, 22, 41]. This last method has also been used in other species such as bananas by comparing DNA from leaf, stem, rhizome and roots [42]. Although all these experiments demonstrate the existence of chimeras in plants and sometimes their crucial impact on agronomical traits, genome wide chimera detection has yet not been possible. Validation of chimeras is also a challenge because we expect low alternative allele frequency since the variant will appears on only one haplotype and one cell layer representing a small proportion of leaf tissue. Sanger sequencing has been found to be limited when alternative allele frequency is under 15–20% [43] but other technologies such as Molecular Inversion Probes (MIP) [44] has proven to be efficient in this particular condition [45]. Widely used in medical programs to detect rare diseases [46–48] it has also been used in plants to detect pathogens [49] or assist in genomic selection [50]. Because of repetitive sequences, it is also difficult to design specific target sequences that only capture the identified SNVs, MIPs along with MIPGEN designing software [51] is efficient on this specific criteria, it also has the advantage of being performant with a small amount of DNA (200ng) which also makes it useful in forensic applications [52, 53]. According to this information MIPs should be an interesting technology for chimera validation.

### Importance of ‘Merlot’ grapevine cultivar

‘Merlot’, which is a cross between ‘Cabernet Franc’ and ‘Magdeleine Noire des Charentes’ [54], is the grape cultivar used in this study. It was first mentioned in south-western France in the late 18th century and expended in Bordeaux area since the middle of 19th century; the impressive spreading of this cultivar in other French regions and worldwide only dates from the 1970s [54]. Today it is the fourth most planted cultivar in the world for table and wine grapes and the second cultivar for wine, cultivated in at least thirty-seven countries on 266 000 ha [55]. It is also the most planted variety in France with 114 578 ha in 2018 [56]. The international success of cv. ‘Merlot’ is mainly explained by the high quality red wines produced in renowned Bordeaux vineyards [57]. This cultivar is also one of the earliest black varieties to be harvested and thus one of the most impacted by climate change. In some areas, cultivating ‘Merlot’ could become inappropriate to produce high quality red wines because of cooked aromas and too high alcohol content [58]. Therefore, exploring ‘Merlot’ genome could open new perspectives to better understand its genetic and physiologic functioning as well as its intravarietal diversity required for clonal preservation and selection. New knowledge on ‘Merlot’ genome and chimeras could also help future grape breeding in order to create improved varieties with a similar fruit phenotype.

Throughout this study we take advantage of the latest sequencing and bioinformatics technologies not only to obtain a whole phased assembly ‘Merlot’ genome but also to contribute to a better understanding of a complex biological phenomena. We used parental sequences of cv. ‘Cabernet Franc’ and ‘Magdeleine Noire des Charentes” to bin ’Merlot’ reads in two groups, assemble the reads per haplotype and build pseudo-molecules. We compared root and leaf sequence to detect periclinal chimeras on each haplotype. We transferred gene annotation from ‘PN40024’ Vcost.v3 [26] to our pseudo-molecules in order to have a functional interpretation of the chimera’s location. Finally, a subset of the chimeras was analysed by MIP in order to validate them with an independent technology.

## Results

### Building pseudo-molecules

DNA samples from ‘Merlot’ lateral roots and leaves and from leaves only for ‘Magdeleine Noire des Charentes’ (maternal) and ‘Cabernet Franc’ (paternal) have been sequenced using Pacific Bioscience Sequel II technology. For each sample between 1.7 and 2.3 million HIFI reads are obtained with an average length of 13 kb and 99.9% phred score accuracy. Taking 500 Mb as *Vitis* genome size we estimate a mean coverage between 47x and 58x according to the sample (Table 2).

**Table 2:** Sequencing quality information for the 4 samples

	Number of sequences	Average length (bp)	Sequence length (bp)	Coverage
Merlot leaf	1 724 709	13 500	46 to 48 499	47x
Merlot root	1 901 058	13 500	47 to 49 695	51x
Cabernet Franc	2 310 232	12 500	48 to 45 849	58x
Magdeleine Noire des Charentes	1 876 162	13 500	44 to 49 698	51x

Sequencing quality is diagnosed for each sample through: the total number of sequences, their average length, their minimal and maximal length and the overall estimated coverage. Merlot leaf and Merlot root refer respectively to the sequencing of DNA extracted from leaves or from lateral roots

Hifiasm trio-binning resolved the raw assembly of each haplotype with high confidence in regards to the statistics presented below (Table 3). Using 500 Mb as the expected genome size of *Vitis vinifera*, the mean N90 of 7,39 Mb with the L90 of 27 allows us to consider these results as being very good quality.

**Table 3:** Assembly quality after trio-binning

Sample name	N50 (Mb)	L50	N90 (Mb)	L90	Number of contigs	Total length (Mb)
Merlot-root-Hap-CF	18.67	10	7.50	26	509	530
Merlot-root-Hap-MG	14.95	12	7.67	30	1 849	586
Merlot-leaf-Hap-CF	21.66	10	8.24	23	206	519
Merlot-leaf-Hap-MG	15.83	12	6.18	31	1 254	550
Average for all samples	17.77	11	7.39	27	955	546

Assembly quality results are defined by N50, L50, N90, L90 statistics. They are completed with the number of contigs assembled and the total length of the assembly. Merlot-root-Hap-CF refers to the root haplotype of Merlot genome transmitted by Cabernet Franc, Merlot-root-Hap-MG, the one transmitted by Magdeleine noire des Charentes, and so on

**Fig. 2:**
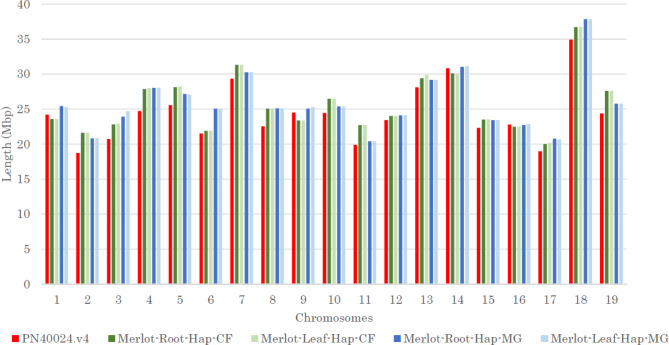
Chromosome length per haplotype compared to PN40024_12X.v4 genome. Chromosome length per haplotype in Mbp for each pseudomolecule built (Merlot-Root-Hap-CF; Merlot-Leaf-Hap-CF; Merlot-Root-Hap-MG; Merlot-Leaf-Hap-MG) against PN40024.v4.

After two successful alignments, first on the PN40024.v4 reference [43] then on the second haplotype (see Material and Methods section), each contig was assigned to its chromosome, their order and orientation were found. In fine, a unique contig to a maximum of 5 were needed to shape chromosomes and between 33 and 47 were used for the whole genome (Table 4). Thus, “Merlot haplotype Cabernet-Franc” (Merlot-hap-CF) is set to 486–490 Mb and “Merlot haplotype Magdeleine Noire des Charentes” (Merlot-hap-MG) to 491 Mb. Because of the sequencing technology and the high performance of long read assembly, we obtain longer chromosomes compared to PN40024.v4. Chromosome lengths are very similar between leaves and roots but trio-binning which correctly phase the assembly, there is a slight difference between Merlot-hap-CF and Merlot-hap-MG (Fig. 2).

**Table 4:** Pseudomolecules characteristics for the haplotypes from both roots and leaves genomes

	Merlot_leaf_CF			Merlot_leaf_MG			Merlot_root_CF			Merlot_root_MG			
	Length (bp)	Contig number	Gene number	Length (bp)	Contig number	Gene number	Length (bp)	Contig number	Gene number	Length (bp)	Contig number	Gene number	
chr01	23,598,264	2	2091	25,285,196	3	2308	23,589,260	2	2090	25,812,159	3	2229	
chr02	21,618,960	2	1711	20,834,059	2	1771	21,612,554	2	1710	20,815,648	2	1773	
chr03	22,910,969	2	1896	23,738,413	3	1853	22,695,420	4	1900	22,863,606	2	1856	
chr04	28,028,847	1	2171	28,067,391	1	2199	27,848,422	1	2172	28,047,555	1	2195	
chr05	28,252,839	3	2354	27,194,848	4	2190	28,133,326	2	2359	27,245,740	5	2197	
chr06	21,902,219	1	1997	25,066,429	2	1901	21,887,941	2	1999	25,064,422	2	1900	
chr07	31,335,031	1	2892	30,285,517	2	2877	31,316,563	1	2889	30,272,463	2	2875	
chr08	25,084,147	2	2260	25,133,383	2	2159	25,068,184	1	2264	25,106,757	2	2163	
chr09	23,385,614	3	1774	25,283,510	3	1789	23,388,363	3	1772	25,079,599	3	1785	
chr10	26,473,290	1	2156	25,356,578	3	2159	26,466,742	1	2159	25,373,432	3	2160	
chr11	20,347,376	2	1571	20,416,243	1	1563	19,944,127	2	1572	20,402,067	1	1569	
chr12	27,064,986	2	2447	24,119,349	4	2347	24,025,996	1	2400	24,096,300	3	2343	
chr13	29,400,470	2	2277	29,181,387	2	2244	29,412,682	3	2277	29,182,803	3	2245	
chr14	30,137,549	1	2577	31,130,771	2	2564	30,138,415	1	2570	31,044,170	1	2545	
chr15	23,518,695	2	1552	23,434,760	2	1832	23,494,571	2	1545	23,422,104	2	1828	
chr16	22,496,135	1	1813	22,861,382	3	1840	22,480,932	3	1815	22,716,031	2	1844	
chr17	20,156,470	2	1552	20,678,794	5	1628	20,368,439	3	1554	20,784,459	4	1628	
chr18	36,729,617	2	3197	37,876,454	1	3161	36,718,487	2	3192	37,875,940	1	3164	
chr19	27,613,142	1	1978	25,782,670	2	1992	27,618,512	2	1979	25,770,078	2	1994	
Total length	490,054,620	33	40,266	491,727,134	47	40,377	486,208,936	38	40,218	490,975,333	44	40,293	
Total + UKN	499,510,259			499,510,259			500,119,474			503,747,540			
chrUn	9,455,639			7,783,125			13,910,538			12,772,207			
%chrUn in total length	1.90%			1.60%			2.80%			2.50%			

For each pseudo-molecule and per chromosome the data shows: the total length, the number of contig necessary to cover the entire chromosome and the number of genes detected. The total length of the pseudo-molecule is found bellow without or with (total + UKN) the unknown chromosome; it is compared with the size of the unknown chromosome (chrUn) which contains all contigs that were not confidently placed on a chromosome

From 1,6% to 2,8% of the assembly can’t be accurately placed in the pseudo-molecule. It is mainly small highly repetitive sequences that are found in different places throughout the genome. This proportion was not attributed to a specific location and couldn’t be associated to a contig.

The results of Benchmarking Universal Single-Copy Orthologs (BUSCO) using embryophyta lineage-specific databases [59] are resumed in Table 5. Results show that up to 98.7% of genes searched are found in the pseudo-molecules and nothing is lost compared to the raw assembly. Duplicated genes are reduced to 1.2–2.2% while missing genes remain around 0.4–0.6%. Therefore, those unplaced contigs were confidently ignored in the further analysis.

**Table 5:** Search for genome completion using BUSCO embryophyta odb 10

	Merlot Hap CF				Merlot Hap MG			
	Leaf		Root		Leaf		Root	
	Raw	Pseudo	Raw	Pseudo	Raw	Pseudo	Raw	Pseudo
Complete	98.7%	98.7%	98.6%	98.5%	98.2%	98.2%	98.2%	98.2%
Complete and single copy	93.2%	96.5%	94.2%	97.1%	96.0%	97.0%	95.1%	97.0%
Complete and duplicated	5.5%	2.2%	4.4%	1.4%	2.2%	1.2%	3.1%	1.2%
Fragmented	0.8%	0.9%	1.0%	0.9%	1.2%	1.2%	1.2%	1.2%
Missing	0.5%	0.4%	0.4%	0.6%	0.6%	0.6%	0.6%	0.6%
Total groups searched	1614							

BUSCO was first performed on raw reads of each haplotype, just after trio-binning (Raw), second BUSCO was done on the pseudomolecules (Pseudo). Complete means that the gene is entirely found, whereas fragmented is not. Single copy means the gene is only found once whereas duplicated means it is found several times. Missing is the percentage of genes expected but not found on the genome. Total groups searched is the number of genes expected

**Fig. 3:**
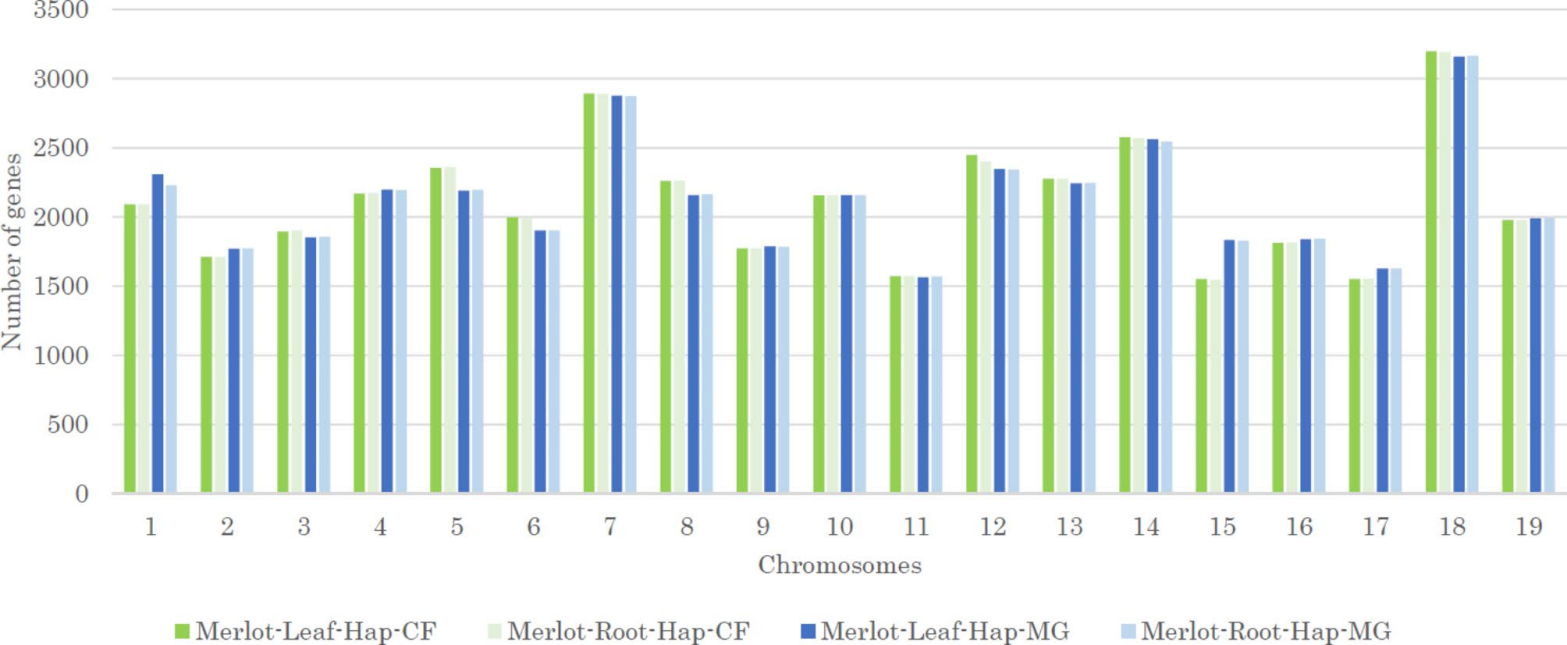
Number of genes per chromosome, for each Merlot sample and each haplotype. Number of gene per chromosome for each pseudomolecule detected by transferred annotation from PN40024 Vcost.v3 using Liftoff

The annotation ‘PN40024’ Vcost.v3 was transferred to each pseudo-molecule using Liftoff tool [60]. In average 95% of the 42 413 genes were positioned throughout the 19 chromosomes with difference of gene numbers between chromosomes as expected (Fig. 3). BUSCO analysis was also performed on protein sequences from ‘PN40024’ Vcost.v3 files and obtained a score of 97,3% complete genes against 95,2% for Merlot-leaf-hap-CF and 95,1% for Merlot-leaf-hap-MG.

### Haplotype comparison

‘Magdeleine Noire des Charentes’ haplotype (Hap-MG) is slightly longer than ‘Cabernet Franc’ haplotype (Hap-CF). Merlot-leaf-hap-MG has 111 more genes than Merlot-leaf-hap-CF (Table 4). All six groups of reads (Cabernet Franc, Magdeleine Noire des Charentes, Merlot-root-hap-CF, Merlot-root-hap-MG, Merlot-leaf-hap-CF, Merlot-leaf-hap-MG) are aligned on Merlot-root-hap-CF pseudo-molecule and DeepVariant is used to perform variant calling (see Material and Methods) [61]. Mapping the reads from both Cabernet Franc haplotypes back on the Merlot-root-hap-CF consensus pseudomolecule allows us to estimate the amount of potential errors in sequencing or assembling. Merlot-root-hap-CF and Merlot-leaf-hap-CF have respectively 3.2k and 3.7k variant sites, mostly in repeated sequences against the pseudo-molecule (Table 6) which are thus potential sequencing errors but could also mean that mosaic mutations appear more frequently in repetitive regions. Mapping the reads from Merlot-MG haplotype and from both ‘Cabernet Franc’ and ‘Magdeleine Noire des Charentes’ lets us accurately compare haplotypes. We identified about 3.5 millions of variants between Merlot-leaf-hap-MG or Merlot-root-hap-MG and Merlot-root-hap-CF pseudo-molecules. These variants are 89% Single Nucleotide Variants (SNV), mainly located in repeated sequences, around 30% are included in a gene region and 5% in a coding region (Table 6).

**Table 6:** Variant calling statistics when reads were aligned on Merlot-root-hap-CF pseudomolecule

Reads aligned on Merlot-root-Hap-CF	Filtered variant sites	% of SNV in filtered variants	% of filtered variants included in repeated Sequences	% of variant sites included in genes	% of variant sites included in coding region
Merlot root hap CF (himself)	3 207	71%	63%	28%	6%
Merlot leaf hap CF	3 731	67%	67%	24%	5%
Merlot leaf hap MG	3 561 942	89%	60%	29%	4%
Merlot root hap MG	3 490 079	89%	60%	29%	4%
Cabernet Franc (paternal)	2 730 140	86%	52%	36%	5%
Magdeleine N. C. (maternal)	4 987 693	89%	57%	31%	5%

This table contains the number of variants found by mapping each packet of reads to the Merlot-Root-Hap-CF pseudomolecule after processing quality filtering, the percentage of these variants that are Single Nucleotide Variant, the percentage of variants included in repeated sequences, the percentage included in genes and the percentage included in a coding region

### Chimera detection

**Fig. 4:**
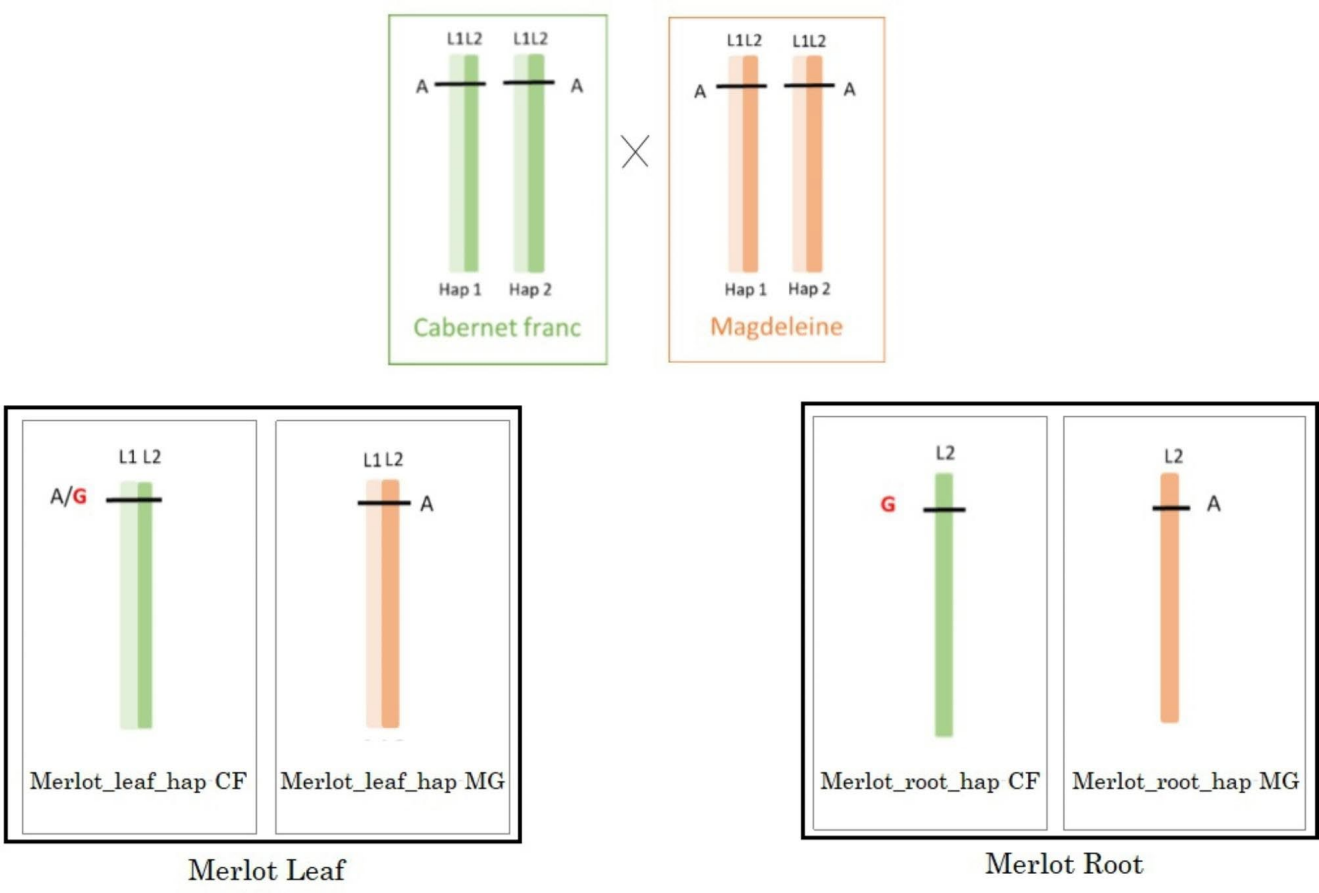
Schematic genetic interpretation of L2 periclinal chimeras in grape cv. ‘Merlot’: Different allele configurations expected for L2 periclinal SNV ‘Merlot’ specific chimeras; a L2 periclinal chimera should be identified in Merlot leaf and root of the same haplotype but not found in either the opposite haplotype nor in parental reads. Each cell layer is represented here as a “stick”, two for leaves and only on for roots. The SNV is represented as either A or G. Cabernet Franc and Magdeleine noire des Charentes leaves are also represented

Periclinal chimeras were detected by variant calling from the alignment of Merlot-leaf-hap-CF reads on Merlot-root-hap-CF pseudo-molecule. As presented in Fig. 1, chimeras can be located on either L1 or L2 cell layers. The comparison of both haplotypes and parental sequences allows to distinguish one case from the other. When Merlot-root-hap-CF reads (L2) and some Merlot-leaf-hap-CF reads (L1 + L2) carry the same allele but all other sequences carry an alternative one, also present in Merlot-leaf-hap-CF reads, we considered that the L2 cell layer has mutated (Fig. 4). On the contrary, when all sequences have the same allele but a variant is confidently detected on Merlot-leaf-hap-CF, we consider the variant allocated to the L1 cell layer. When these mutations are confirmed by all root reads and are present only in a subset but not in all leaf reads, it means that the entire L2 meristem cell layer carries the mutation and can be called periclinal chimeras. To increase confidence in detection, we focused on haplotype specific chimeras when no variant is found in reads of the opposite haplotype. We also chose to only select ‘Merlot’ specific chimeras and therefore excluded variants if parental reads were heterozygous. Grapevine DNA also has a lot of repeated sequences known to evolve more rapidly (e.g. microsatellites, transposable elements). In this study we focused on periclinal chimera that are located in non-repeated sequences because they are more stable and less prone to mapping errors. Only SNVs were kept. The work was executed on each haplotype separately. In total, 51 positions match the requirements on Merlot-hap-CF, and 53 on Merlot-hap-MG (Table 7).

**Table 7:** Number of chimeras per haplotype and per cell layer

	Merlot Haplotype CF			Merlot Haplotype MG		
	L1	L2	L1 + L2	L1	L2	L1 + L2
SNV, Non-repeated sequences Merlot specific chimeras	37	14	**51**	36	17	**53**
Number of chimeras in non coding gene body	15	4	**19**	11	5	**16**
Number of chimeras in coding region	6	3	**9**	6	1	**7**

Chimeras are found by comparing root and leaves DNA. Results are shown per haplotype and per location on the cell layer: either L1 or L2, L1 + L2 is the sum of both. First line gives the total number of position detected, second line is the number of position included in a gene body region but not in a coding region and the last line is the number included in a coding region inside the gene

Respectively 37 and 36 are found on L1 cell layer for Merlot-Hap-CF and Merlot-Hap-MG and 14 and 17 respectively on L2 cell layer. A total of 19 and 16 on each haplotype are located in a gene region, 9 and 7 chimeras are in a coding region. The exact position of the chimeras in the genome, the nucleotide and the number of reads for each allele, the type of chimera and location in coding region are presented in Tables 8 and 9.

**Table 8:** SNVs Chimeras found on Merlot-hap-CF

CELL LAYER	Chr	Pos	Merlot Leaf Hap CF		Merlot Root Hap CF		Merlot Leaf Hap MG		Merlot Root Hap MG		Cabernet		Magdeleine		Annotation of Merlot root hap CF pseudomolecule	
L1	chr01	622,811	G/T	*16/7*	G	*22*	G	*3*	G	*6*	G	*64*	G	*59*	No	
L1	chr01	10,384,263	G/A	*28/9*	G	*31*	G	*28*	G	*23*	G	*60*	G	*52*	Gene	
L1	chr02	7,380,049	T/C	*22/6*	T	*23*	T	*14*	T	*19*	T	*63*	T	*43*	CDS	
L1	chr04	23,822,006	C/G	*20/8*	C	*19*	C	*21*	C	*32*	C	*67*	C	*48*	Gene	
L1	chr04	25,819,229	A/G	*12/5*	A	*26*	A	*29*	A	*43*	A	*57*	A	*65*	No	
L1	chr05	22,934,305	C/T	*22/7*	C	*21*	C	*16*	C	*30*	C	*56*	C	*25*	Gene	
L1	chr05	23,010,268	A/G	*7/31*	A	*33*	A	*29*	A	*19*	A	*71*	A	*45*	No	
L1	chr05	25,416,770	C/T	*15/5*	C	*19*	C	*22*	C	*25*	C	*48*	C	*57*	No	
L1	chr06	3,621,220	T/C	*10/8*	T	*32*	T	*2*	T	*4*	T	*64*	T	*65*	No	
L1	chr06	12,834,474	A/G	*20/10*	A	*16*	A	*25*	A	*19*	A	*59*	A	*56*	No	
L1	chr07	8,599,845	C/T	*22/8*	C	*30*	C	*26*	C	*33*	C	*65*	C	*44*	Gene	
L1	chr07	12,188,378	A/G	*23/8*	A	*29*	A	*29*	A	*33*	A	*46*	A	*44*	No	
L1	chr08	20,988,006	C/T	*17/11*	C	*29*	C	*24*	C	*35*	C	*65*	C	*43*	CDS	
L1	chr08	22,606,874	T/C	*18/5*	T	*37*	T	*14*	T	*9*	T	*64*	T	*31*	No	
L1	chr10	16,298,623	A/G	*22/13*	A	*30*	A	*21*	A	*28*	A	*54*	A	*51*	Gene	
L1	chr11	7,012,825	G/A	*23/10*	G	*30*	G	*30*	G	*26*	G	*46*	G	*53*	Gene	
L1	chr12	8,907,103	C/A	*17/8*	C	*23*	C	*23*	C	*17*	C	*22*	C	*28*	CDS	
L1	chr12	16,700,578	A/G	*27/14*	A	*37*	A	*17*	A	*16*	A	*52*	A	*35*	Gene	
L1	chr13	1,078,880	A/G	*22/10*	A	*29*	A	*22*	A	*25*	A	*67*	A	*53*	No	
L1	chr13	19,791,949	A/T	*25/8*	A	*28*	A	*22*	A	*29*	A	*61*	A	*48*	No	
L1	chr13	23,444,751	A/T	*23/9*	A	*30*	A	*28*	A	*29*	A	*71*	A	*63*	Gene	
L1	chr14	2,105,195	A/C	*24/7*	A	*25*	A	*17*	A	*23*	A	*64*	A	*52*	No	
L1	chr14	8,620,581	T/C	*24/7*	T	*24*	T	*21*	T	*21*	T	*53*	T	*42*	Gene	
L1	chr14	15,844,322	C/T	*19/7*	C	*12*	C	*27*	C	*28*	C	*33*	C	*45*	No	
L1	chr15	19,297,087	C/T	*27/8*	C	*33*	C	*26*	C	*33*	C	*53*	C	*53*	CDS	
L1	chr15	20,162,161	G/A	*23/11*	G	*31*	G	*20*	G	*35*	G	*45*	G	*70*	CDS	
L1	chr15	20,298,163	C/A	*16/8*	C	*22*	C	*16*	C	*24*	C	*43*	C	*38*	Gene	
L1	chr16	18,511,980	T/A	*19/9*	T	*35*	T	*23*	T	*32*	T	*57*	T	*59*	No	
L1	chr17	8,087,306	C/A	*14/7*	C	*23*	C	*13*	C	*15*	C	*35*	C	*47*	No	
L1	chr17	9,516,464	C/G	*18/8*	C	*32*	C	*27*	C	*16*	C	*75*	C	*66*	Gene	
L1	chr17	12,223,344	A/C	*18/10*	A	*32*	A	*9*	A	*2*	A	*37*	A	*22*	Gene	
L1	chr18	4,105,052	T/C	*16/6*	T	*24*	T	*29*	T	*29*	T	*64*	T	*43*	Gene	
L1	chr18	5,367,698	C/A	*23/6*	C	*34*	C	*33*	C	*41*	C	*79*	C	*63*	Gene	
L1	chr18	11,379,480	G/T	*22/9*	G	*37*	G	*27*	G	*26*	G	*58*	G	*30*	Gene	
L1	chr18	12,312,986	C/A	*12/4*	C	*27*	C	*26*	C	*32*	C	*73*	C	*26*	No	
L1	chr18	21,684,668	G/A	*32/10*	G	*27*	G	*4*	G	*7*	G	*50*	G	*27*	No	
L1	chr19	6,420,354	C/T	*9/7*	C	*22*	C	*34*	C	*30*	C	*55*	C	*66*	CDS	
L2	chr04	26,224,776	T/C	*21/7*	T	*34*	C	*28*	C	*29*	C	*73*	C	*56*	No	
L2	chr05	24,987,737	C/T	*22/11*	C	*31*	T	*26*	T	*28*	T	*70*	T	*61*	No	
L2	chr06	17,243,628	T/A	*20/10*	T	*44*	A	*20*	A	*38*	A	*94*	A	*58*	No	
L2	chr07	10,337,296	G/A	*11/5*	G	*22*	A	*26*	A	*31*	A	*58*	A	*53*	No	
L2	chr10	8,445,101	T/A	*17/12*	T	*24*	A	*26*	A	*30*	A	*72*	A	*47*	Gene	
L2	chr11	9,424,215	A/G	*19/8*	A	*27*	G	*28*	G	*30*	G	*62*	G	*57*	Gene	
L2	chr12	22,268,147	A/T	*22/9*	A	*21*	T	*11*	T	*12*	T	*46*	T	*27*	CDS	
L2	chr14	3,806,097	T/C	*9/6*	T	*30*	C	*21*	C	*28*	C	*35*	C	*29*	No	
L2	chr15	13,526,354	A/G	*23/8*	A	*29*	G	*26*	G	*35*	G	*49*	G	*60*	CDS	
L2	chr17	3,559,600	C/T	*20/10*	C	*32*	T	*16*	T	*23*	T	*56*	T	*49*	Gene	
L2	chr17	7,610,259	C/T	*15/5*	C	*24*	T	*30*	T	*27*	T	*64*	T	*46*	Gene	
L2	chr18	12,292,200	G/A	*19/8*	G	*35*	A	*28*	A	*35*	A	*64*	A	*77*	No	
L2	chr19	468,364	G/T	*25/10*	G	*32*	T	*32*	T	*29*	T	*74*	T	*66*	CDS	
L2	chr19	17,464,909	A/G	*19/6*	A	*21*	G	*10*	G	*4*	G	*23*	G	*15*	No	

For each chimera, the cell layer is identified, the chromosome and the exact location is given according to the reference Merlot-root-Hap-CF pseudo molecule. Then for each sample the allele(s) are completed by the number of reads confirming the nucleotide. If there is only one nucleotide, all reads of this haplotype converge, when two alleles are found the number of reads supporting each one is respectively given by the corresponding numbers. For the first position (chr 01, position 622 811), 16 reads have “G” nucleotide and 7 have “T” nucleotide. In the last column the position is either included in a coding region inside a gene (CDS), included in a gene but not in a coding region (gene) or not included in either (No).

**Table 9:** SNVs Chimeras found on Merlot-hap-MG

Cell Layer	Chr	Position	Merlot Leaf Hap MG		Merlot Root Hap MG		Merlot Leaf Hap CF		Merlot Root Hap CF		Cabernet		Magdeleine		Annotation of Merlot root hap MG
L1	chr02	3,007,441	A/G	*26/7*	A	*33*	A	*24*	A	*27*	A	*24*	A	*32*	No
L1	chr03	3,419,274	T/A	*12/7*	T	*33*	T	*23*	T	*16*	T	*66*	T	*47*	CDS
L1	chr04	27,055,780	A/T	*18/7*	A	*27*	A	*22*	A	*35*	A	*64*	A	*38*	Gene
L1	chr05	8,794,703	C/T	*17/8*	C	*37*	C	*9*	C	*14*	C	*20*	C	*41*	Gene
L1	chr05	8,797,586	C/A	*23/9*	C	*31*	C	*8*	C	*3*	C	*19*	C	*27*	Gene
L1	chr05	12,819,049	T/A	*16/7*	T	*24*	T	*25*	T	*32*	T	*50*	T	*31*	Gene
L1	chr05	18,874,107	C/T	*22/10*	C	*23*	C	*24*	C	*26*	C	*47*	C	*52*	Gene
L1	chr07	16,367,184	C/T	*23/7*	C	*37*	C	*16*	C	*20*	C	*54*	C	*63*	No
L1	chr08	13,911,900	C/A	*16/9*	C	*33*	C	*15*	C	*22*	C	*44*	C	*63*	CDS
L1	chr08	18,990,379	A/T	*16/7*	A	*10*	A	*20*	A	*17*	A	*53*	A	*31*	No
L1	chr09	2,285,675	G/T	*23/7*	G	*34*	G	*22*	G	*24*	G	*61*	G	*51*	No
L1	chr09	7,569,730	A/G	*16/8*	A	*21*	A	*3*	A	*3*	A	*35*	A	*43*	No
L1	chr09	11,451,290	G/A	*20/8*	G	*30*	G	*28*	G	*23*	G	*30*	G	*32*	No
L1	chr10	2,278,377	C/A	*20/13*	C	*29*	C	*24*	C	*29*	C	*81*	C	*71*	Gene
L1	chr10	5,337,832	A/G	*16/10*	A	*24*	A	*11*	A	*12*	A	*44*	A	*48*	CDS
L1	chr10	10,968,103	A/C	*18/5*	A	*36*	A	*27*	A	*33*	A	*65*	A	*38*	Gene
L1	chr10	22,333,673	T/A	*18/6*	T	*28*	T	*28*	T	*14*	T	*85*	T	*63*	Gene
L1	chr11	10,058,232	A/C	*8/12*	A	*33*	A	*19*	A	*21*	A	*43*	A	*41*	No
L1	chr11	10,357,099	T/C	*15/5*	T	*27*	T	*16*	T	*13*	T	*42*	T	*49*	No
L1	chr11	13,563,841	T/C	*10/6*	T	*23*	T	*4*	T	*6*	T	*16*	T	*16*	No
L1	chr11	13,732,865	T/C	*21/4*	T	*33*	T	*16*	T	*27*	T	*61*	T	*68*	CDS
L1	chr11	13,734,413	A/C	*22/5*	A	*31*	A	*20*	A	*27*	A	*64*	A	*69*	Gene
L1	chr13	15,741,877	A/T	*14/7*	A	*18*	A	*27*	A	*24*	A	*86*	A	*74*	No
L1	chr13	18,415,015	A/G	*17/9*	A	*24*	A	*3*	A	*4*	A	*25*	A	*27*	Gene
L1	chr13	22,993,757	T/A	*16/14*	T	*33*	T	*31*	T	*37*	T	*53*	T	*56*	No
L1	chr13	24,830,909	A/T	*18/10*	A	*20*	A	*19*	A	*32*	A	*65*	A	*43*	Gene
L1	chr14	5,457,647	T/A	*22/6*	T	*19*	T	*3*	T	*6*	T	*17*	T	*28*	No
L1	chr14	30,136,904	A/G	*13/9*	A	*30*	A	*9*	A	*14*	A	*60*	A	*54*	No
L1	chr15	2,385,711	C/T	*10/6*	C	*16*	C	*2*	C	*2*	C	*26*	C	*21*	No
L1	chr16	6,369,473	C/T	*20/6*	C	*24*	C	*6*	C	*7*	C	*24*	C	*21*	No
L1	chr16	15,506,287	C/T	*22/7*	C	*29*	C	*14*	C	*22*	C	*62*	C	*27*	No
L1	chr16	17,466,134	C/T	*24/8*	C	*28*	C	*16*	C	*17*	C	*45*	C	*67*	No
L1	chr17	7,800,769	G/A	*13/5*	G	*22*	G	*20*	G	*14*	G	*49*	G	*21*	CDS
L1	chr17	16,802,779	C/T	*26/9*	C	*37*	C	*35*	C	*33*	C	*76*	C	*50*	No
L1	chr17	20,480,355	C/T	*10/4*	C	*32*	C	*25*	C	*28*	C	*49*	C	*39*	CDS
L2	chr03	17,695,770	G/A	*23/7*	G	*24*	A	*22*	A	*18*	A	*44*	A	*66*	Gene
L2	chr05	1,753,844	T/C	*10/8*	T	*24*	C	*17*	C	*11*	C	*55*	C	*43*	No
L2	chr05	5,349,008	A/G	*19/9*	A	*23*	G	*28*	G	*35*	G	*63*	G	*75*	Gene
L2	chr07	20,018,317	G/A	*23/7*	G	*25*	A	*31*	A	*27*	A	*64*	A	*66*	No
L2	chr08	5,971,148	A/G	*20/10*	A	*30*	G	*26*	G	*17*	G	*71*	G	*56*	Gene
L2	chr08	8,665,113	A/C	*18/6*	A	*26*	C	*32*	C	*28*	C	*36*	C	*51*	No
L2	chr08	10,405,377	C/T	*17/4*	C	*27*	T	*28*	T	*30*	T	*71*	T	*77*	No
L2	chr08	16,511,687	A/G	*23/5*	A	*37*	G	*23*	G	*20*	G	*50*	G	*60*	No
L2	chr08	18,095,503	C/T	*24/8*	C	*33*	T	*26*	T	*17*	T	*60*	T	*52*	No
L2	chr10	3,422,488	G/T	*17/5*	G	*30*	T	*31*	T	*23*	T	*28*	T	*98*	No
L2	chr11	1,451,292	C/T	*22/8*	C	*32*	T	*32*	T	*43*	T	*80*	T	*63*	No
L2	chr12	7,859,048	A/C	*22/11*	A	*19*	C	*24*	C	*30*	C	*66*	C	*58*	Gene
L2	chr12	14,034,710	T/G	*15/8*	T	*16*	G	*13*	G	*25*	G	*50*	G	*24*	No
L2	chr13	25,521,690	A/G	*25/7*	A	*26*	G	*24*	G	*29*	G	*58*	G	*51*	Gene
L2	chr14	16,511,432	T/A	*10/8*	T	*21*	A	*22*	A	*22*	A	*42*	A	*51*	No
L2	chr15	20,391,775	T/G	*20/5*	T	*30*	G	*37*	G	*31*	G	*76*	G	*50*	CDS
L2	chr17	17,822,477	C/T	*20/7*	C	*25*	T	*12*	T	*19*	T	*47*	T	*62*	No

For each chimera, the cell layer is identified, the chromosome and the exact location is given according to the reference Merlot-root-Hap-MG pseudo molecule. Then for each sample the allele(s) are completed by the number of reads confirming the nucleotide. If there is only one nucleotide, all reads of this haplotype converge, when two alleles are found the number of reads supporting each one is respectively given by the corresponding numbers. For the first position (chr 02, position 1 351 259), 15 reads have G nucleotide and 6 have T. In the last column the position is either included in a coding region inside a gene (CDS), included in a gene but not in a coding region (gene) or not included in either (No).

### Validation of chimeras by MIPs

Out of the 104 positions identified as chimeras, MIPGEN software was able to design a ligation and extension probe for 95 target regions. MIPseq was performed on Merlot_leaf and Merlot_root samples. Amplification was obtained for 86 positions but only 32 had enough depth to compare both samples (Table 10 and Additional files 2 and 3). 22 positions have the expected alleles on each sample and validate with enough depth PacBio results. 8 positions have the expected alleles but also have a single read that is unexpected which makes them ambiguous. One position is invalid because an allele is missing on Merlot leaf sample (chr3-3419274 on MG haplotype). Finally, one position is classified as ambiguous (chr10-22333673 on MG haplotype) because it has both alleles on leaves and roots. This doesn’t necessarily invalid the existence of the chimera but makes it unclear on which cell layer it is located on.

**Table 10:** Chimera validation with molecular inversion probes sequencing

				Merlot 343 Leaf			Merlot 343 Root			Validation conclusion
	Cell layer	Chro-mosome	Position	Expected alleles according to PacBio	Mip validation		Expected alleles according to PacBio	Mip validation		
Chimeras on Merlot haplotype ‘Cabernet Franc’	L1	chr04	23 822 006	C/G	C/G	25/10	C	C	11/0	Validated
	L1	chr05	25 416 770	C/T	C/T	125/24	C	C	45/0	Validated
	L1	chr08	22 606 874	T/C	T/C	16/6	T	T	13/0	Validated
	L1	chr10	16 298 623	A/G	A/G	101/10	A	A	25/0	Validated
	L1	chr12	8 907 103	C/A	C/A	34/2	C	C	10/0	Validated
	L1	chr12	16 700 578	A/G	A/G	47/15	A	A/G	11/1	1 unexpected read
	L1	chr14	8 620 581	T/C	T/C	190/16	T	T/C	73/1	1 unexpected read
	L1	chr14	15 844 322	C/T	C/T	71/4	C	C	19/0	Validated
	L1	chr15	20 162 161	G/A	G/A	11/1	G	G/A	11/1	1 unexpected read
	L1	chr18	4 105 052	T/C	T/C	102/6	T	T	15/0	Validated
	L2	chr17	7 610 259	C/T	C/T	6/29	C/T	C/T	4/6	Validated
	L2	chr18	12 292 200	G/A	G/A	56/67	G/A	G/A/C	16/27/1	1 unexpected read
Chimeras on Merlot Haplotype ‘Magdeleine Noire des Charentes’	L1	chr03	3 419 274	T/A	T	75	T	T	16	Invalid
	L1	chr05	8 797 586	C/A	C/A	35/5	C	C	18	Validated
	L1	chr05	12 819 049	T/A	T/A	61/5	T	T	21	Validated
	L1	chr07	16 367 184	C/T	C/T	172/16	C	C	49	Validated
	L1	chr08	13 911 900	C/A	C/A/G	67/13/1	C	C	36	1 unexpected read
	L1	chr09	7 569 730	A/G	A/G	20/1	A	A	10	Validated by only 1 read
	L1	chr10	2 278 377	C/A	C/A	110/18	C	C	47	Validated
	L1	chr10	22 333 673	T/A	T/A	53/26	T	T/A	18/6	Confusion on cell layer
	L1	chr11	10 357 099	T/C	T/C	26/10	T	T	10	Validated
	L1	chr13	22 993 757	T/A	T/A	17/2	T	T/C	10/1	1 unexpected read
	L1	chr14	30 136 904	A/G	A/G	25/5	A	A/T	10/1	1 unexpected read
	L1	chr17	7 800 769	G/A	G/A	73/4	G	G	21	Validated
	L1	chr17	20 480 355	C/T	C/T	123/19	C	C	42	Validated
	L2	chr07	20 018 317	G/A	G/A	75/112	G/A	G/A	18/45	Validated
	L2	chr08	8 665 113	A/C	A/C	64/132	A/C	A/C	13/23	Validated
	L2	chr08	18 095 503	C/T	C/T	51/58	C/T	C/T	17/28	Validated
	L2	chr12	14 034 710	T/G	T/G	46/71	T/G	T/G	20/15	Validated
	L2	chr14	16 511 432	T/A	T/A	62/179	T/A	T/A	52/51	Validated
	L2	chr15	20 391 775	T/G	T/G	38/58	T/G	T/G	8/13	Validated
	L2	chr17	17 822 477	C/T	C/T	32/27	C/T	C/T	12/22	Validated

Results of molecular inversion probes sequencing is shown per haplotype; the location of the previously identified chimera is detailed. For both samples, first column contains the alleles found by PacBio sequencing, the second one the alleles revealed by MIP and the third one the number of reads that support each allele separated by “/” symbol. Valid positions have the expected alleles according to PacBio results and ambiguous or invalid have unexpected alleles.

## Discussion

The combination of long reads high quality sequencing, trio binning using parental sequences, and a long read assembler gives the opportunity to resolve accurately phased assembly. This new ‘Merlot’ genome encompasses a total length of about 500 Mbp, and was constructed with only 33 to 47 contigs. Some chromosomes were resolved with a single contig, while others needed up to 5. The number of contigs for each chromosome for the 4 haplotypes were however different, therefore not linked to a specific chromosome, but most probably to sequence depth. Chromosome lengths and gene number were slightly different between both haplotypes. The gene numbers may however be underestimated since no de novo annotation was done. In the objective towards the definition of grape pangenome, precise *de novo* annotation of ‘Merlot’ genome should be performed. According to Shumate and Salzberg [60], Liftoff can accurately transfer 99.9% of the genes when working intra-species. Here, PN40024 reference genome for *Vitis vinifera* has been used to transfer genes on a *Vitis vinifera* cultivar. Because of this intra specific design it is expected that liftoff accurately transferred most of the genes, this is confirmed by the close results of BUSCO analysis performed on protein sequences, 97% of complete genes for PN40024 against 95% for Merlot pseudomolecules. Moreover, the program works by aligning the reference genes on the target sequence, so although it can’t identify new genes the mapping correspondence can be considered accurate.

The pseudomolecules obtained in this study were compared to existent whole genome sequencing data available in the literature, information is compiled in “Additional file 1”. The closest genome found in terms of assembly quality is the ‘Cabernet-Sauvignon’ assembly [32]. However, taking advantage of the newest technologies the pseudo molecules obtained in this work have longer assemblies (~ 490 Mb), longer average sequence lengths (~ 26 Mb), longer maximum lengths (~ 37 Mb), longer N50 (~ 25 Mb) and higher Busco scores (~ 98%) with less missing genes (~ 0.5%). Moreover, these technologies are time saving as the complete assembly and pseudomolecule building can be done in a couple of days. Liftoff offers a fast tool to transfer annotation, 96% of the genes from ‘PN40024’ Vcost.v3 were successfully replaced on all four pseudo-molecules allowing a functional interpretation of the results.

### Advantage of having parental reads

The originality of this study was to not only sequence the cultivar of interest but also its parents. Parental sequences have allowed to discriminate ‘Merlot’ reads from each haplotype. Each haplotype was then assembled independently, as if we had two homozygous individuals. This step increases confidence in haplotype comparison statistics. Haplotype differences in a single individual (~ 3.5 million of variant sites) are similar to what has already been reported [41]. The pseudomolecule used as reference for the variant calling is the Merlot-root-hap-CF which shares half of the ‘Cabernet Franc’s DNA. This explains why 2.7 million variants are detected for ‘Cabernet Franc’ (difference for only one haplotype) whereas ‘Magdeleine Noire des Charentes’ displayed 4.9 million variants, both haplotypes being different. In addition, being able to retrace parental origin could make it possible to know what agronomic character comes from each parent which increases possibilities in breeding and cultivar improvement. Considering the difference between both ‘Merlot’ haplotypes, ~ 60% variants are located on repeated sequences, ~ 30% are located in gene regions and ~ 6% in coding regions. These numbers align with the apportionment of each of one in the genome [25]. This suggests that the variants are not preferentially located in coding or repeated sequences. However, this doesn’t fit with previous publications on ‘Pinot Noir’ [62] or ‘Nebbiolo’ [63] that found more variations in coding regions between both haplotypes. This could be a specificity of ‘Merlot’ haplotypes since differences in variation rates have already been noticed between ‘Nebbiolo’ and ‘Zinfandel’ or it could also be explained by trio binning technology that allows to rebuild each haplotype more accurately and therefore have a better appreciation of the comparison.

### Chimera identification and their impact on the phenotype

Until now, chimera detection was only possible with PCR sequencing when three alleles were found on the same locus or by dissecting tissues derived from different meristem cell layers as cited above. However, a genome wide screening of chimeras was not yet possible with these methods. Throughout this study we show that quantity and high quality sequencing, long reads, trio binning and organ comparison and strong selection open new doors in chimera detection. Around 3 000 variants were found when mapping Merlot-leaf-hap-CF and Merlot-root-hap-CF reads to the Merlot-root-hap-CF pseudomolecule, these are mainly SNVs (67–71%). The variants identified by mapping Merlot-root-hap-CF reads on the consensus pseudomolecule most certainly correspond to sequencing, trio binning or assembly errors but it is also possible that some of these variants are sectorial chimeras and only located in a few cells. To detect chimeras, we remove these 3 000 positions. In addition, we focused on variants outside repeated sequences which are easier to map and more likely to be stable during evolution and less prone to errors. We also focused on variants that meet periclinal chimera definition because they are the most stable. They indeed meet very specific conditions but they are also the fewest. Nevertheless, the very selective criteria applied allow us to confidently identify these variants as being chimeras. It is not excluded that other types of chimeras exist but were not selected in this work. Indeed, a mutation can be present in a few cells of one or both cell layers and appear as a variant site but it would need extra experiments to truly validate them.

Similar amounts of SNV periclinal chimeras were found on each haplotype (51 and 53). These results seem to mean that they appear randomly and at the same frequency on both haplotypes. Among those, 70% correspond to mutations on L1 cell layer and 30% on L2 cell layer. Some sequencing errors detected on leaf samples and not on roots could explain this difference between L1 and L2 although such difference in frequency could also make sense because L1 cell layer is located on the surface of leaves and is more exposed to UV radiation. Moreover, L2 cell layer produces gametes and are probably more protected [64]. Validation on independent reads is needed the support this last theory.

The consequences of a chimera depend on its position on the genome, in our study 33% are located in a gene body region and 15% are located in a coding region and could modify the protein which can be perceived on the phenotype (Tables 7, 8, 9, 11 and 12). Although our data confirms this possibility, the phenomena appears to be a rare event.

**Table 11:** Description of the periclinal chimeras on Merlot-Hap-CF located in a gene

Chr	Position on Merlot Root Hap CF	Cell layer	Coding region	Gene orientation	REF Codon	REF Amino Acid	ALT Codon	ALT Amino Acid	Gene name	LOC	NAME	
chr 01	10,384,263	L1	gene						Vitvi01g00875	LOC100263854	disease resistance protein TAO1	
chr 02	7,380,049	L1	CDS	reverse	A**A**C	N	A**G**C	S	Vitvi02g00607.CDS16	LOC100260794	uncharacterized	
chr 04	23,822,006	L1	gene						Vitvi04g02171.t01	LOC104879118	protein FREE1	
chr 05	22,934,305	L1	gene						Vitvi05g01344.t01	LOC100248036	eukaryotic translation initiation factor 3 subunit H	
chr 07	8,599,845	L1	gene						Vitvi07g00711	LOC100251541	WD repeat-containing protein 26	
chr 08	20,988,006	L1	CDS	reverse	AA**G**	K	AA**A**	K	Vitvi08g01573.CDS1	LOC100259003	caffeic acid 3-O-methyltransferase	
chr 10	8,445,101	L2	gene						Vitvi10g01813.t01	LOC104880497	uncharacterized	
chr 10	16,298,623	L1	gene						Vitvi10g01120.t01	LOC100252938	lysine-specific demethylase JMJ18	
chr 11	7,012,825	L1	gene						Vitvi19g00471.t01			
chr 11	9,424,215	L2	gene						Vitvi11g00777.t01	LOC100267388	autophagy-related protein 11	
chr 12	8,907,103	L1	CDS	forward	CT**C**	L	CT**A**	L	Vitvi12g02494.CDS1	LOC100251126	myelin transcription factor 1-like protein	
chr 12	16,700,578	L1	gene						Vitvi12g01666.t01	LOC100245109	protein FAM135B	
chr 12	22,268,147	L2	CDS	reverse	**T**AG	STOP	**A**AG	K	Vitvi12g02051.CDS3	LOC104881		
chr 13	23,444,751	L1	gene						Vitvi13g01459.t01	LOC100263567	lowering time control protein FY [ *Vitis vinifera*	
chr 14	8,620,581	L1	gene						Vitvi14g00503	LOC100250122	phosphoribosylaminoimidazole carboxylase, chloroplastic	
chr 15	13,526,354	L2	CDS	forward	A**A**C	N	A**G**C	S	Vitvi15g00455.CDS2			
chr 15	19,297,087	L1	CDS	forward	T**C**C	S	T**T**C	F	Vitvi15g00839.CDS9	LOC100260889	homeobox-leucine zipper protein ANTHOCYANINLESS 2	
chr 15	20,162,161	L1	CDS	reverse	AC**C**	T	AC**T**	T	Vitvi15g00907.CDS5	LOC100245390	rho GTPase-activating protein 5 [ *Vitis vinifera*	
chr 15	20,298,163	L1	gene						Vitvi00g01964.t01			
chr 17	3,559,600	L2	gene						Vitvi17g00302.t01	LOC100267388	autophagy-related protein 11	
chr 17	7,610,259	L2	gene						Vitvi17g01488.t01	LOC100251937	zinc finger protein 346	
chr 17	9,516,464	L1	gene						Vitvi17g00813.t01	LOC100250198	serine/threonine-protein kinase ATM	
chr 17	12,223,344	L1	gene						Vitvi17g01006.t01	LOC100255285	callose synthase 10	
chr 18	4,105,052	L1	gene						Vitvi18g00368.t01	LOC100247288	acetyl-CoA carboxylase 1	
chr 18	5,367,698	L1	gene						Vitvi18g00496	LOC100255806	WD and tetratricopeptide repeats protein 1	
chr 18	11,379,480	L1	gene						Vitvi18g02778.t01	LOC100247127	purple acid phosphatase 2	
chr 19	468,364	L2	CDS	reverse	G**C**G	A	G**A**G	E	Vitvi19g01801.CDS6	LOC100255520	probable leucine-rich repeat receptor-like serine/threonine-protein kinase At3g14840	
chr 19	6,420,354	L1	CDS	reverse	GT**G**	V	GT**A**	V	Vitvi19g00471.CDS1			

All chimeras included in a gene are shown in this table. Their exact location on Merlot-Root-Hap-CF and the cell layer are given. If the chimera is located in a coding region it is marked “CDS” otherwise it is “gene”. When the position is in a coding region, the gene orientation either reversed or forward is given with the reference codon and amino acid, the nucleotide carrying the mutation is written in bold. The alternative sections show the impact of the mutation on the codon and the amino acid. Three last columns allow gene identification through gene name, LOC code and name.

**Table 12:** Description of the periclinal chimeras on Merlot-Hap-MG located in a gene region

Chr	Position on Merlot Root Hap MG	Cell layer	Coding region	Gene orientation	REF Codon	REF Amino Acid	ALT Codon	ALT Amino Acid	Gene name	LOC	NAME	
chr 03	3,419,274	L1	CDS	reverse	**A**TC	I	**T**TC	F	Vitvi03g01484.CDS2	LOC100243450	isoflavone reductase-like protein	
chr 03	17,695,770	L2	gene						Vitvi03g01777			
chr 04	27,055,780	L1	gene						Vitvi04g01695.t01	LOC100259141	GDP-mannose transporter GONST1	
chr 05	5,349,008	L2	gene						Vitvi05g00464	LOC100261408	chloride channel ClC6	
chr 05	8,794,703	L1	gene						Vitvi05g00751.t01			
chr 05	8,797,586	L1	gene						Vitvi05g00751.t01			
chr 05	12,819,049	L1	gene						Vitvi05g00975.t01	LOC100242520	dihydroxy-acid dehydratase, chloroplastic	
chr 05	18 874 107	L1	gene						Vitvi05g01197.t01			
chr 08	5,971,148	L2	gene						Vitvi08g00297	LOC100245232	uncharacterized	
chr 08	13,911,900	L1	CDS	reverse	C**G**G	R	C**T**G	L	Vitvi08g00910.CDS1	LOC100249911	uncharacterized LOC100249911	
chr 10	2,278,377	L1	gene						Vitvi00g01047.t01	LOC100853350	sister chromatid cohesion protein PDS5 homolog B	
chr 10	5,337,832	L1	CDS	forward	AG**A**	R	AG**G**	R	Vitvi10g00473.CDS1	LOC100258824	putative nuclear RNA export factor SDE5	
chr 10	10,968,103	L1	gene						Vitvi10g00847.r01	LOC104880520	uncharacterized LOC104880520	
chr 10	22,333,673	L1	gene						Vitvi10g01425.t01			
chr 11	13,732,865	L1	CDS	forward	AG**T**	S	AG**C**	S	Vitvi11g00979.CDS8	LOC100246466	DEAD-box ATP-dependent RNA helicase 10	
chr 11	13,734,413	L1	gene						Vitvi11g00979.t01	LOC100246466	DEAD-box ATP-dependent RNA helicase 10	
chr 12	7,859,048	L2	gene						Vitvi12g00621	LOC100263032	5' exonuclease Apollo	
chr 13	18,415,015	L1	gene						Vitvi13g01203.r01	LOC104881233	uncharacterized LOC104881233	
chr 13	24,830,909	L1	gene						Vitvi13g02403.t01	LOC100261918	annexin D4-like	
chr 13	25,521,690	L2	gene						Vitvi13g02429	LOC100244776	uncharacterized	
chr 15	20,391,775	L2	CDS	forward	**T**CA	S	**G**CA	A	Vitvi15g00944.exon15	LOC100255258	uncharacterized	
chr 17	7,800,769	L1	CDS	forward	T**G**C	C	T**A**C	Y	Vitvi17g00653.CDS1			
chr 17	20,480,355	L1	CDS	reverse	**G**TA	V	**A**TA	I	Vitvi17g01265.CDS1			

All chimeras included in a gene are shown in this table. Their exact location on Merlot-Root-Hap-MG and the cell layer are given. If the chimera is located in a coding region it is marked “CDS” otherwise it is “gene”. When the position is in a coding region, the gene orientation either reversed or forward is given with the reference codon and amino acid, the nucleotide carrying the mutation is written in bold. The alternative sections show the impact of the mutation on the codon and the amino acid. Three last columns allow gene identification through gene name, LOC code and name

MIP sequencing allowed to validate with confidence a subset of positions which makes chimera detection through hifi PacBio long reads and trio-binning reliable. However, MIP sequencing results overall did not have the depth expected compared to what is described in the literature leading to the loss of more than half of the positions tested. This means that either MIP target region design or laboratory protocol should be optimised. Having unexpected alleles only supported by a single read makes conclusion ambiguous but could be due to sequencing errors, mutation induced by PCR or it could be due to the higher sensitivity of the MIP technology to detect rare mutations. It appears that each technology has its own pros and cons and only a cross result between two sequencing technologies can bring a high confidence in the detection of the chimeras. However, PacBio technology seems trustworthy to detect SNVs on one hand and also makes it possible to determine on which haplotype and which cell layer chimeras are located on.

Throughout this study we have made a specific focus on single nucleotide variant because they are more stable. Yet some essential functions or characteristics of grapevine such as berry colour can be altered by structural variants [20], studying these types of variants would also be of interest.

Chimeras are rare but they can have a strong impact on phenotype. If they are identified and selected, they can lead to a new cultivar as it has already been reported with ‘Pinot Gris’. In a less obvious evolution, perennial plants propagated over centuries only through cuttings, chimeras are most likely to accumulate over time and could slowly induce genetic diversity among the cultivar. By continuously selecting the best plant to fit specific characteristics, breeders increase their chance to select and propagate useful chimeras. When chimeras are stable and conserved through several generations of cuttings, they could also be used to trace and identify clonal lineage. Since we have developed a tool for revealing chimeras, it would be interesting to analyse the presence of a subset of the chimeric mutations in different ‘Merlot’ clone 343 plants in order to check how stable these chimeras might be. For grapevine, clonal identification is an important issue because no low cost and rapid test can guarantee clonal origin, although it is the economic unit used today. Clonal lineage is only done by human traceability which can contain errors especially after a long period of time.

## Conclusion

Through this study, whole genome DNA sequence was obtained using the latest genomic technologies and bioinformatics tools. Hifi long read sequencing, trio-binning, long read assembler, have all together allowed to obtain high quality, haplotype resolved pseudo-molecules. In addition, repeat masker tools, mapping and deep variant calling opened new possibilities in chimera detection. By comparing root and leaf samples and through severe selection it has been possible to identify hundred chimeras based on SNVs on both haplotypes. MIP validation has confirmed the presence of these chimeras. Other types of chimeras could be present, but we were not able to identify them. A functional interpretation was done through transferred annotation. Actual genomic tools open new doors in chimera detection, representing opportunities for perennial plant breeding. In addition, this high quality ‘Merlot’ genome, could also open new perspectives such as structural variants identifications, but could also serve as a basis for a study of intra-varietal variability for this cultivar.

## Materials and methods

### DNA sequencing

‘Magdeleine Noire des Charentes’ leaves were harvested from INRAE Vassal-Montpellier grape collection (Marseillan, FRANCE), while ‘Merlot’ clone 343 leafs and roots as well as ‘Cabernet Franc’ were harvested from IFV collection, Domaine de l’Espiguette (Grau du Roi, FRANCE). Two young leaves about 10 cm wide were collected, carefully rolled over and placed in a 13 ml tube. Secondary lateral roots from the same ‘Merlot’ clone 343 plant were also collected on the same day. This plant was not grafted and was destined to be pulled out which made it possible to collect its roots. All samples were conserved in a -80°C freezer until the DNA extraction process. DNA was extracted following the Tip 100 Qiagen Genomic kit with slight modifications. Lysis was performed 3 hours at 50°C on 0.5 g of ground plant material with 9.5 ml of G2 buffer supplemented with 1% PVP-40, 19 µl of RNase A and 500 µl of proteinase K. After tip filtrations, DNA was precipitated with isopropanol, centrifuged 15 min à 5000 g, washed with Ethanol 70° and re-suspended in 50 µl of TE buffer. DNA quality and high molecular weight were controlled. DO 260/280 ratio between 1.8 and 2.0 and DO 260/230 ratio between 2.0 and 2.2 were confirmed and an Agilent Genomic DNA Screen Tape was performed. Fifteen µg of high quality DNA were then used to carry out the sequencing. Samples were sequenced using Single Molecule Real Time PacBio SEQUEL II hifi long reads at INRAE Clermont-Ferrand GENTYANE platform (France).

### Assembly and building pseudo-molecules

DNA consensus call sequences obtained under BAM format were converted to fastq using bam2fastq tool from SMRTLink v9.0.0 PacBio library. The HIFI sequencing DNA quality was verified using FastQC version 0.11.7.

**Fig. 5:**
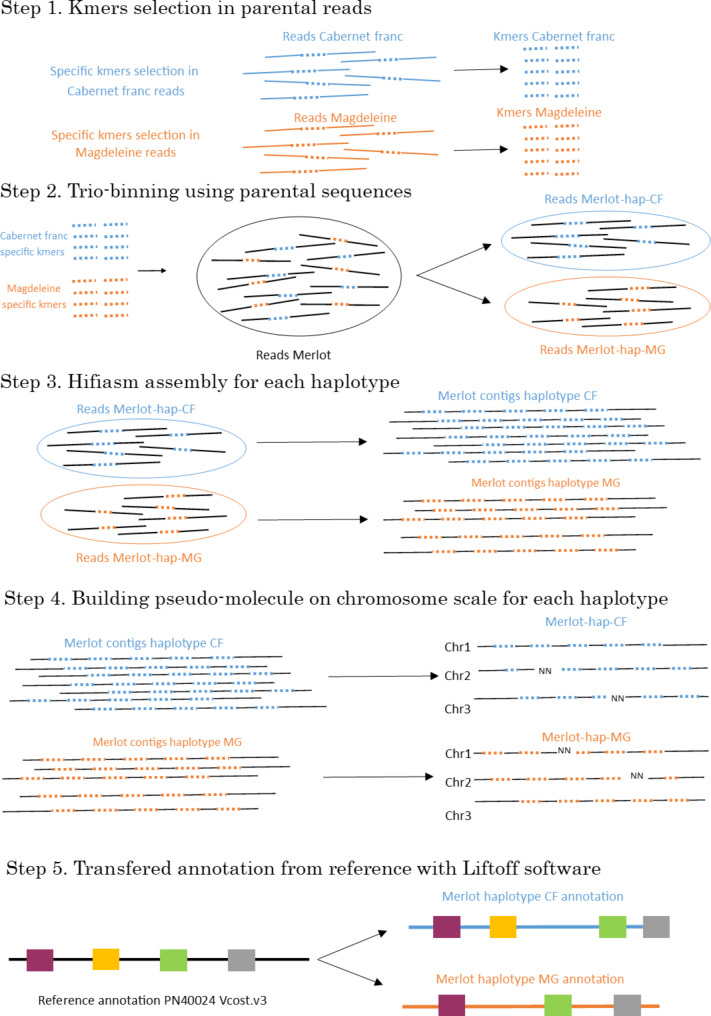
Bioinformatic workflow applied in this study. The workflow is described step by step. Step 1 is the kmers specific selection on each parent, these are variable size sequences that allow to specifically recognize reads from on parent. In step 2 these kmers are used to sort child reads out in two haplotypes that are each specific to one parent. If reads can’t be attributed to one parent, they are considered to be in both. Step 3 is the assembly of several reads into contigs for each haplotype. Step 4 is the building of pseudomolecules using multiple alignments. Step 5 is the transfer of annotation from the reference genome to the pseudomolecules

Figure 5 illustrates the whole bioinformatics workflow to build pseudo-molecules and transfer annotation. Paternal and maternal kmers were identified using the parental reads with yak-0.1 software. The outputs were then used in hifiasm-0.13 with default parameters to bin ‘Merlot’ long reads and assemble both haplotypes. This was done on both organs (leaf and root).

For each haplotype, contigs were aligned on PN40024.v4 using minimap2 version 2.17 [65]. Best contigs alignments were used to build an AGP file and from there reconstruct each pseudo-molecule. In order to refine the pseudo-molecules, we then reexecuted the same process starting with an alignment of each haplotype on the other previously reconstructed.

The embryophyta_odb10 lineage from BUSCO 5.3.1 software was carried out in genome mode to estimate the completeness of all assemblies [59]. BUSCO was also performed on protein sequences using “prot” option, protein sequences were obtained from the pseudomolecules by using gffread tool version 0.12.6 with default parameters.

Liftoff 1.6.1 tool with default parameters was used to transfer the annotation of PN40024 Vcost.v3 reference genome to the pseudomolecules [60].

### Chimera detection

Reads were mapped on Merlot-root-hap-CF and Merlot-root-hap-MG pseudomolecules with Minimap2 version 2.17 [65] with the option –x map-hifi and variant calling was performed with DeepVariant software version 1.1.0 [66] using PacBio model and default parameters. Finally filtering variants was done with vcftools 0.1.16 version [67].

Chimera detection was processed by filtering vcf from Merlot-leaf on Merlot-root pseudomolecule. We only conserved variants with more than 10 depth coverage, “PASS” quality flag and genotype quality (GQ) over 20. Non homozygous positions on all other sequences were excluded. Repeated sequences were identified by building a specific ‘Merlot’ library with repeatmodeler/2.0.2a-bin [68] and then using repeatmasker/4.1.1 software [69], and all chimeras in repeated sequences were excluded. Both repeatmodeler and repeatmasker were used with default parameters. Only single nucleotide variants were kept. Finally, Tables 7, 8 and 9 were manually checked site per site by visualisation in Integrative Genome Viewer (IGV 2.12.3) that allows a larger overview of the region on several samples [70]. These sites were crossed with the annotation file with intersect Bed function of BEDtools/2.30.0 [71] and Table 10 and 11. were completed.

### Chimera validation

MIPGEN software [51] was used to design mips specific target regions previously identified as chimeras with following parameters: tag sizes 0.8 to introduce UMI (Unique Molecular Identifiers) to filter out duplicate reads and PCR errors, minimum ligature length 20, extension minimum length 16, arm length sums 36, 37, 38, 39, 40, minimum capture size 120, maximum capture size 150 and trf option was activated. DNA samples were adjusted in quantity using the previous DNA extraction and used in adapted MIP library protocol previously described [72] with some modifications. 100 ng of DNA template was added to a hybridization mix together with the oligo MIP pool (final concentration of 0.025 pM per probe) in 0.85x Ampligase buffer (Epicentre). Mix was incubated in a thermal cycler at 95 °C for 10 min, followed by a 60 °C cycle overnight. Products were mixed with dNTPs (Jena Bioscience, 15 pM), Betaine (Sigma-Aldrich, 375 mM), NAD+ (New England, Biolabs 1 mM), additional Ampligase buffer (0.75×), Ampligase (Epicentre, 1.25 U) and Klentaq (New England Biolabs, 0.16U). Mixture was incubated at 56 °C for 60 min followed by 72 °C for 20 min. Enzymatic digestion of linear probes was performed at 37 °C for 2 h, followed by 80 °C for 20 min by adding Exonuclease I (New England Biolabs, 8 U) and Exonuclease III (New England Biolabs, 50 U). Final product was amplified using Q5 Hot start High-Fidelity DNA Polymerase (New England Biolabs, 8 U) with different index combinations. PCR cycling conditions were an initial denaturation step for 2 min at 98 °C, followed by 20 cycles of 30 s at 98 °C, 20 s at 60 °C, and 20 s at 72 °C. PCR Samples were pooled and clean up using AMPureXP beads (BeckmanCoulter) at 0.8× ratio. Samples were sequenced in 2 × 150 bp paired end mode using a MiSeq (Illumina) platforms with custom sequencing primers. UMI were extracted from obtained reads using umi_tools version 1.1.4 extract [73] with --extract-method = string and --bc-pattern = NNNNNNNNNNNN. Adapters were trimmed using cutadapt version 3.5 [74] with following parameters: -q 30 -m 100 -e 0.10 -a ACACTACCGTCGGATCGTGCGTGT -A CTTCAGCTTCCCGATTACGGATCTCGTATG. SNP calling was done using process_reseq from VCFhunter version 2.2.0 with -s acefg option [75]. Finally, variant calling file was filtered when depth was below 10 for at least one sample.

## Electronic supplementary material

Below is the link to the electronic supplementary material.

**Additional file 1**: Additional_file_1.xls. Comparative data between whole genome sequences already published and the pseudomolecules built in this study.

**Additional file 2**: Additional_file_2.xls. PacBio and MIP data for each chimera found on Merlot-hap-MG.

**Additional file 3**: Additional_file_3.xls. PacBio and MIP data for each chimera found on Merlot-hap-CF.

## Data Availability

Raw reads of PacBio sequencing are available on the European Nucleotide Archive repository, under the project named PRJEB59893: https://www.ebi.ac.uk/ena/browser/view/PRJEB59893. Contigs, AGP file, chromosome scale assembly and annotations transferred from PN40024 VCost.v3 with liftoff are available on Recherche Data Gouv: 10.57745/OJ07SN. PN40024 sequence and annotation used in this study are available on INTEGRAPE platform, https://integrape.eu/resources/genes-genomes/genome-accessions/. Merlot clone 343 is available at INRAE, domaine de Vassal, under the code 0Mtp2399.

## Abbreviations

RT PCR: Real-Time Polymerase Chain Reaction
SSR: Single Sequence Repeat
SNV: Single nucleotide Variant
